# Supplementary material for: Ubiquitin and TFIIH-stimulated DDB2 dissociation drives DNA damage handover in nucleotide excision repair
Source: Nat Commun. 2020 Sep 28;11:4868. doi: 10.1038/s41467-020-18705-0 (PMC7522231; doi:10.1038/s41467-020-18705-0)
Supplement: Supplementary file 1 — Supplementary Information [file 41467_2020_18705_MOESM1_ESM.pdf]

Supplementary Information

Ubiquitin and TFIIH-stimulated DDB2 dissociation drives DNA damage handover in nucleotide excision repair

Cristina Ribeiro-Silva[1], Mariangela Sabatella[1,2], Angela Helfricht[1], Jurgen A. Marteijn[1], Arjan F. Theil[1], Wim Vermeulen[1,*], Hannes Lans[1,*]

1. Department of Molecular Genetics, Oncode Institute, Erasmus MC, University Medical Center Rotterdam, Dr. Molewaterplein 40, 3015 GD, Rotterdam, The Netherlands

2. Present Address: Princess Máxima Center for pediatric oncology, Heidelberglaan 25, 3584 CS, Utrecht

* Correspondence and requests for materials should be addressed to:

HL (w.lans@erasmusmc.nl) or WV (w.vermeulen@erasmusmc.nl)

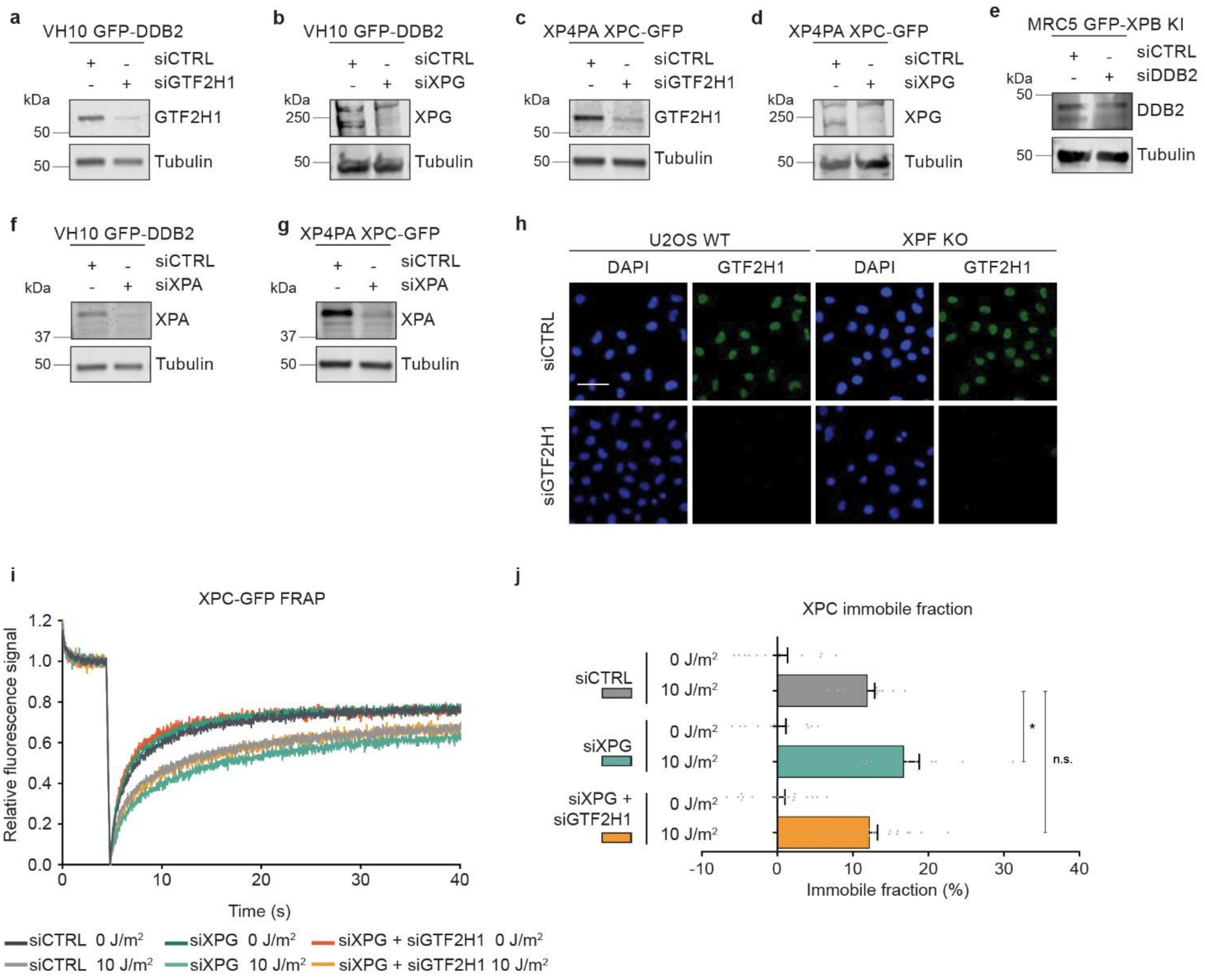

Supplementary Figure 1. Knockdown efficiencies and XPC-GFP FRAP after XPG and GTF2H1 knockdown. (**a-g**) Immunoblot analyses of total cell lysates demonstrating the efficiency of siRNA-mediated protein depletion with siRNAs against GTF2H1, XPG, XPA and DDB2 in the indicated cell lines and probed with antibodies against the respective proteins. Tubulin was used as loading control. (**h**) Immunofluorescence (IF) images stained with antibodies against GTF2H1, demonstrating the efficiency of GTF2H1 siRNA-mediated depletion in U2OS WT and XPF KO cells. Scale bar: 50 µm. Knockdown was verified in each experiment in which siRNA was used. (**i**) FRAP analysis of XPC mobility in mock or UV-C irradiated (10 J/m^2) XP4PA cells stably expressing XPC-GFP and transfected with control (CTRL), XPG and GTF2H1 siRNAs. (**j**) Percentage of XPC-GFP immobile fraction in XP4PA cells treated with control (CTRL), XPG and GTF2H1 siRNAs, determined by FRAP analysis as depicted in (i). Graphs and FRAP curves depict mean & S.E.M. of, respectively, n=12, 12, 12, 12, 17, 22 cells from two independent experiments. * $P < 0.05$, n.s., non-significant, relative to siCTRL control 10 J/m^2, analyzed by unpaired, two-tailed t-test (adjusted for multiple comparison, see methods). Source data are provided as a Source Data file.

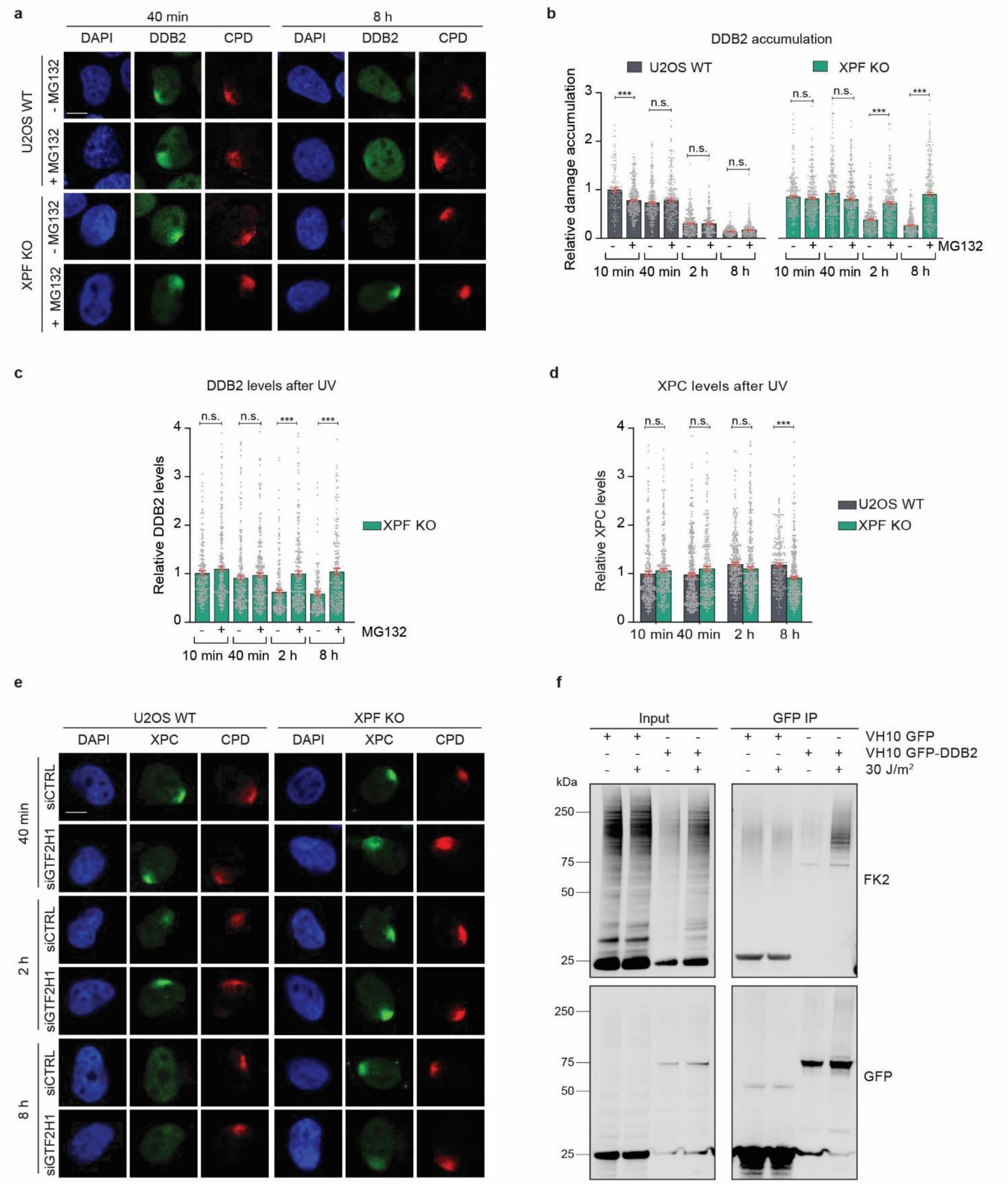

Supplementary Figure 2. XPC and DDB2 recruitment to LUD. (**a**) Representative IF images of endogenous DDB2 accumulation at LUD in U2OS WT and XPF KO cells in the absence or presence of proteasome inhibitor MG132. 1 h before LUD induction, 50 µM MG132 was added to cells, which were fixed 40 min and 8 h after local UV irradiation (60 J/m^2) through a microporous membrane (8 µm). Scale bar: 5 µm. (**b**) Quantification of endogenous DDB2 accumulation at LUD in U2OS WT and XPF KO cells, as described in (a). DDB2 accumulation was normalized to the nuclear background and to U2OS WT (– MG132) 10 min, which was set to 1.0. Mean & S.E.M. of, respectively, n=127,

246, 263, 166, 300, 238, 238, 172, 229, 281, 227, 211, 276, 310, 273, 242 cells from three independent experiments. **c**) Total DDB2 protein levels in XPF KO cells 10 min, 40 min, 2 h and 8 h after LUD induction, as described in (a), determined by measuring total nuclear fluorescent signal intensities in nuclei and normalized to XPF KO (– MG132) 10 min, which was set to 1.0. Mean & S.E.M. of, respectively, n=229, 281, 227, 211, 276, 310, 276, 242 cells from two independent experiments. (**d**) Total XPC protein levels determined by measuring total nuclear fluorescent signal intensities in nuclei such as depicted in Fig. 2d and normalized to U2OS WT 10 min, which was set to 1.0. Mean & S.E.M. of, respectively, n=305, 412, 338, 227, 275, 309, 382, 338 cells from five independent experiments. (**e**) Representative IF images of endogenous XPC recruitment to LUD (marked by CPD staining) 40 min, 2 h and 8 h after UV-C (60 J/m^2) through a microporous membrane (8 µm) in U2OS WT cells treated with control (CTRL) or GTF2H1 siRNAs. Quantified in Fig. 3c. Scale bar: 5 µm. (**f**) Immunoblot analysis of DDB2 ubiquitylation in VH10 cells stably expressing GFP-DDB2 or GFP alone, before or 15 min after UV-C irradiation (30 J/m^2). GFP and GFP-DDB2 were immunoprecipitated (IP) using GFP beads. Total cell lysates (Input) and IP fractions (GFP IP) samples were analyzed for ubiquitylation and DDB2, using anti-ubiquitin (FK2) and GFP antibodies, respectively. The experiment was repeated independently with similar results. *** $P < 0.001$, n.s., non-significant, analyzed by one-way ANOVA (see methods). Source data are provided as a Source Data file.

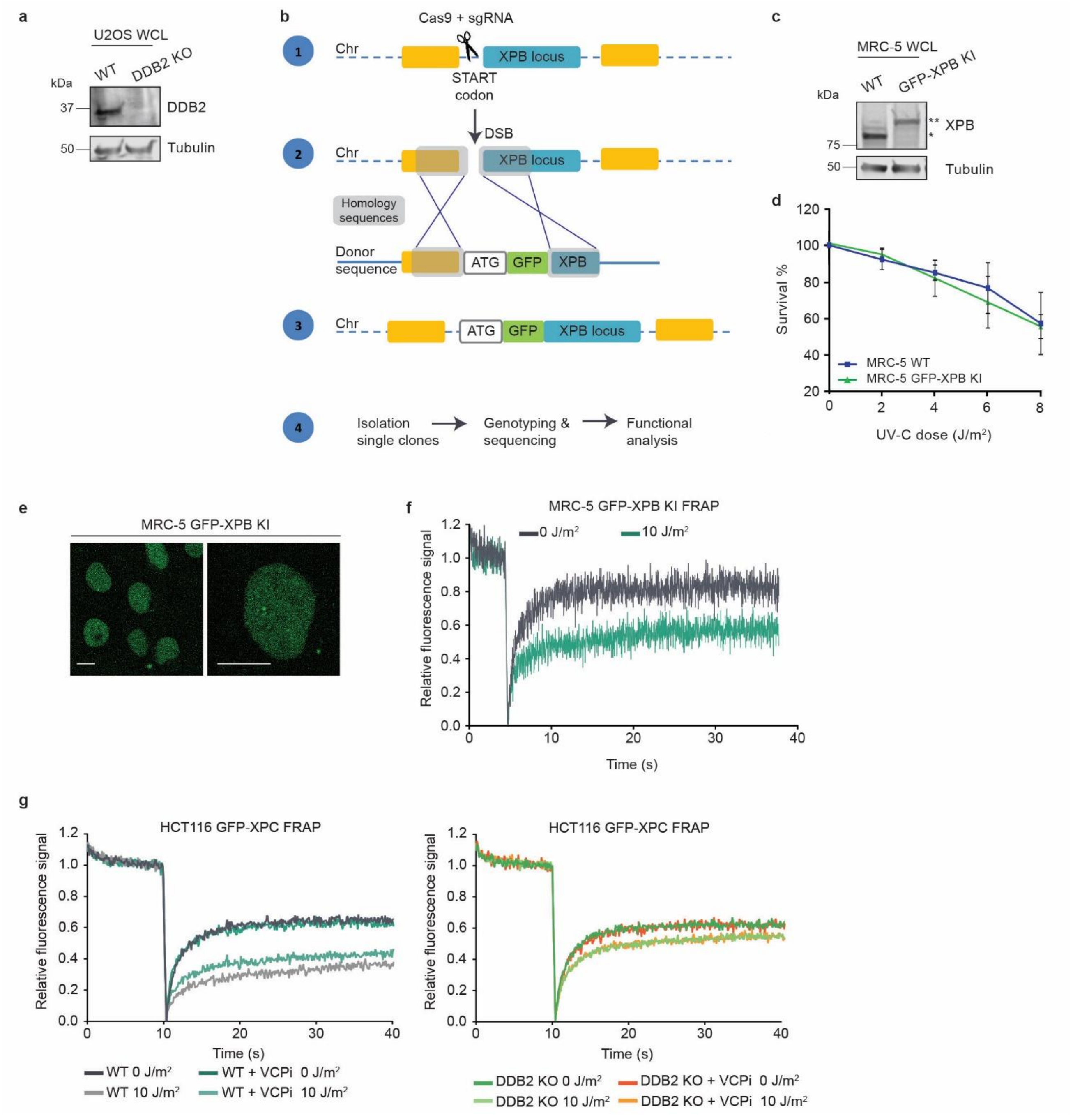

Supplementary Figure 3. Generation of DDB2 KO and GFP-XPB knock-in cell lines. (**a**) Whole cell lysate (WCL) immunoblot analysis of U2OS WT and DDB2 KO cells with DDB2 antibody. Tubulin was used as loading control. (**b**) Schematic representation of the GFP knock-in (GFP-KI) strategy in the *XPB* locus using CRISPR-Cas9 homology-directed repair, generating cells with stable and endogenous expression of GFP-tagged XPB. (**c**) Whole cell lysate (WCL) immunoblot analysis of WT MRC-5 cells and GFP-XPB KI clone with XPB antibody. Wild-type XPB is marked with * and GFP-XPB with **. Tubulin was used as loading control. (**d**) Colony survival after UV-C irradiation of WT MRC-5 cells and GFP-XPB KI. Survival was plotted as the percentage of colonies obtained after treatment compared to the mean number of colonies from the mock-treated cells, set as 100%. Mean & S.E.M. of three independent experiments, each performed in triplicate. (**e**) MRC-5 GFP-XPB KI live cell confocal images showing nuclear GFP-XPB expression. Scale bars: 15 µm (left and right). (**f**)

Characterization of endogenous XPB mobility in MRC-5 GFP-XPB KI cells before and immediately after UV irradiation (10 J/m^2) using FRAP analysis. GFP-XPB fluorescence recovery was measured in a strip across the nucleus after bleaching and normalized to the average pre-bleach intensity (1.0). (**g**) FRAP analysis of endogenously GFP-tagged XPC mobility in HCT116 cells before and after UV irradiation (10 J/m^2), in the presence (WT) and absence (KO) of DDB2. Cells were mock treated or incubated with VCPi (10 µM) 1 h before UV irradiation. GFP-XPC fluorescence recovery was measured in a strip across the nucleus for 30 s after bleaching and normalized to the average pre-bleach intensity (1.0). Source data are provided as a Source Data file. Experiments shown in (a), (c) and (e) were repeated independently with similar results.

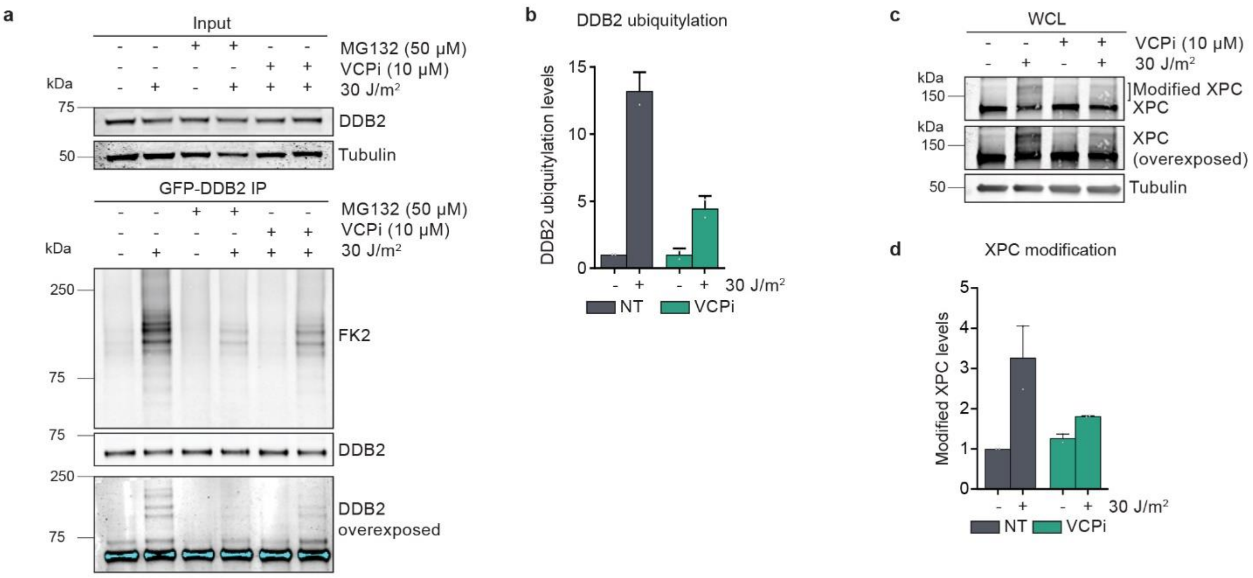

Supplementary Figure 4. MG132 and VCPi inhibit UV-induced ubiquitylation of DDB2 and XPC. (**a**) Immunoblot analysis of DDB2-ubiquitylation levels in VH10 cells stably expressing GFP-DDB2, mock-treated or treated with 50 μM of proteasome inhibitor MG132 or 10 μM of VCPi, 30 min and 1 h before UV (30 J/m^2), respectively. GFP-DDB2 IP fractions and total cell lysates (Input) were immunoblotted and probed with antibodies against ubiquitin (FK2) and DDB2. Tubulin was used as loading control. (**b**) Quantification of ubiquitin (FK2) levels in the GFP-DDB2 IP fraction depicted in (a). Ubiquitin levels were normalized to DDB2 and to the mock-treated condition, which was set to 1.0. * P< 0.05, relative to non-irradiated condition. Mean of two independent experiments. (**c**) Immunoblot analysis of XPC ubiquitylation in U2OS cells mock-treated or treated with VCPi (10 μM), 1 h before UV (30 J/m^2). Whole cell lysates (WCL) were analyzed by immunoblotting using an antibody against XPC. Tubulin staining was used as loading control. (**d**) Quantification of ubiquitin-modified XPC in WCL of U2OS, depicted in (c). Modified XPC levels were normalized to Tubulin and to the mock-treated condition, which was set to 1.0. Mean of two independent experiments. Source data are provided as a Source Data file.

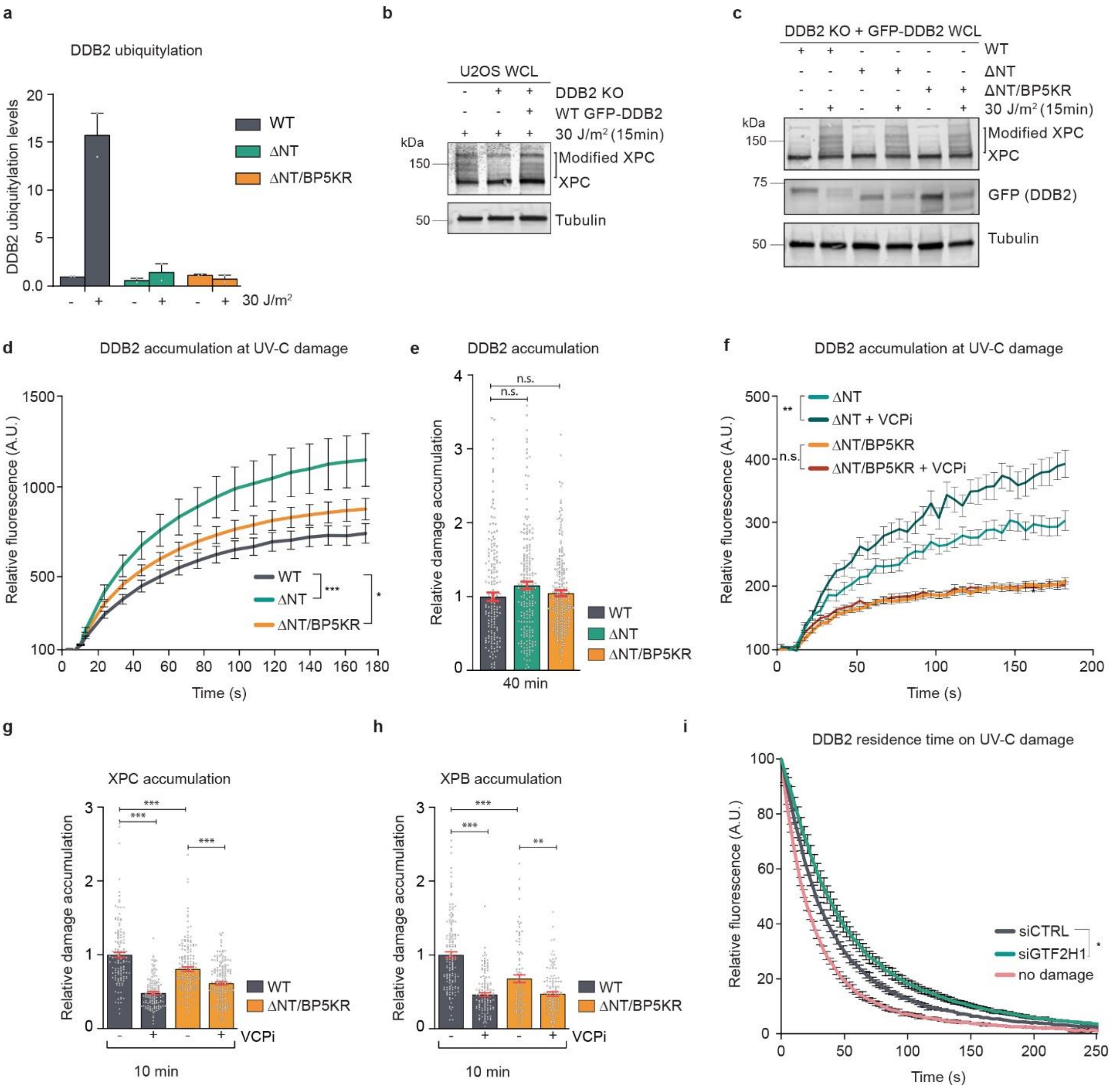

Supplementary Figure 5. Analysis of WT and mutant GFP-DDB2 cell lines. (**a**) Quantification of ubiquitin (FK2) levels in the GFP-DDB2 IP fraction revealed by immunoblotting and shown in Fig. 6c. Ubiquitin levels were normalized to DDB2 and to the mock-treated condition, which was set to 1.0. Mean of two independent experiments. (**b**) Immunoblot analysis of XPC ubiquitylation in whole cell lysate (WCL) of U2OS WT and DDB2 KO cells with or without stable expression of WT GFP-DDB2, prepared 15 min after UV (30 J/m^2) and analyzed with an antibody against XPC. Tubulin staining was used as loading control. (**c**) Immunoblot analysis of XPC ubiquitylation in WCL of U2OS DDB2 KO cells with stable expression of WT, ΔNT, or ΔNT/BP5 GFP-DDB2, stained with XPC, DDB2 and Tubulin antibodies. (**d**) Accumulation of WT, ΔNT and ΔNT/BP5KR GFP-DDB2 variants at LUD, induced with a 266 nm UV-C laser (coupled to a Leica SP5 microscope), measured in real-time by confocal imaging. Pre-damage relative fluorescence intensity was set to 100% (t=0). Mean & S.E.M. from > 30 cells per condition from three independent experiments. * $P < 0.05$, *** $P < 0.001$, analyzed by unpaired, two-tailed t-test (adjusted for multiple comparison, see methods) regarding the last accumulation time point. (**e**) Quantification of ectopic DDB2 accumulation at LUD 40 min after UV

irradiation (60 J/m^2) through a microporous membrane (8 µm) in U2OS DDB2 KO cells stably complemented with WT, ΔNT or ΔNT/BP5KR GFP-DDB2. Normalization to the nuclear background and WT GFP-DDB2 after UV-C, which was set to 1.0. Mean & S.E.M. of > 163 cells from three independent experiments. n.s., non-significant, analyzed by one-way ANOVA (**f**) Accumulation of ΔNT and ΔNT/BP5KR GFP-DDB2 variants at LUD, induced with a 266 nm UV-C laser (coupled to a SP8 Leica microscope), measured in real-time by confocal imaging. Cells were pre-treated with VCPi 1 h before imaging. Pre-damage relative fluorescence intensity was set to 100% (t=0). Mean & S.E.M. from > 36 cells per condition. ** P < 0.01, n.s., non-significant, analyzed by ROC curve analysis (see methods). ΔNT GFP-DDB2 accumulation is likely still influenced by VCPi due to the presence of ubiquitylated lysines that are mutated in the ΔNT/BP5KR GFP-DDB2 mutant. (**g, h**) Quantification of endogenous (**g**) XPC and (**h**) XPB accumulation at LUD in U2OS DDB2 KO cells stably complemented with WT or ΔNT/BP5KR GFP-DDB2, normalized to the nuclear background and to mock-treated WT GFP-DDB2, which was set to 1.0. Mock- or VCPi- treated cells were fixed 10 min after LUD induction. Mean & S.E.M. of, respectively, n=129, 142, 164, 170 cells for XPC and n=146, 137, 128, 159 cells for XPB from two independent experiments. ** P < 0.01, *** P < 0.001, analyzed by one-way ANOVA (see methods) (**i**) iFRAP analysis of WT GFP-DDB2 dissociation from non-damaged DNA (pink line) and from local UV DNA damage after control (CTRL, grey line) or GTF2H1 (green line) siRNA treatment in VH10 cells stably expressing wild type GFP-DDB2. Fluorescence was measured over time, normalized to background and to fluorescence levels before bleaching. Mean & S.E.M. of > 30 cells per condition from three independent experiments. * P < 0.05, analyzed by ROC curve analysis (see methods). Source data are provided as a Source Data file.

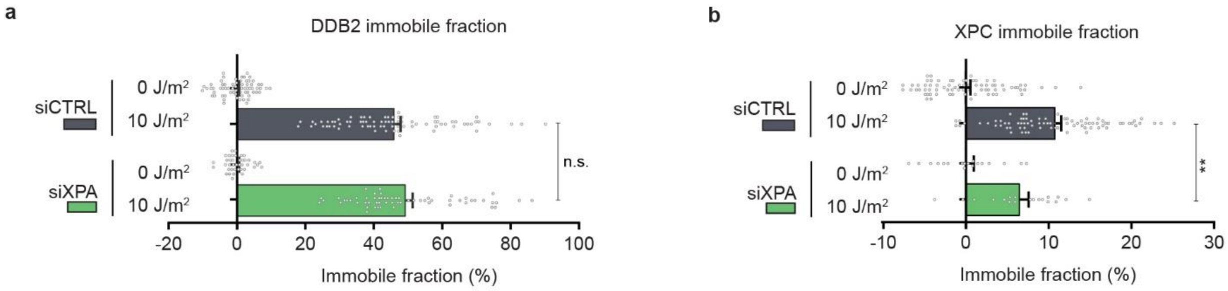

Supplementary Figure 6. Differential regulation of damage sensing proteins DDB2 and XPC by XPA. (**a**) Percentage of GFP-DDB2 immobile fraction in VH10 fibroblasts treated with control (CTRL) or XPA siRNAs, determined from FRAP analyses as described in Fig. 1. Percentage immobile fraction represents the ratio between the average recovered fluorescence intensity of UV- and mock-treated cells, over the last 10 s of the measurements, as explained in the methods. (**b**) Percentage of XPC-GFP immobile fraction in XP4PA cells treated with control (CTRL) or XPA siRNAs, determined by FRAP analysis as described in Fig. 1. Graphs in (a) and (b) depict mean & S.E.M. of > 30 cells for each condition from three independent experiments. ** $P < 0.01$, n.s., non-significant, analyzed by unpaired, two-tailed t-test (adjusted for multiple comparison, see methods). Source data are provided as a Source Data file.

Supplementary Table 1. Primary Antibody list and working dilutions.

Source, Reference	Antibody	Dilutions	
		Immunoblotting	Immunofluorescence
Abcam, ab181136	DDB2	1/1000	1/1000
Bethyl, A301-121A	XPC	1/2000	1/2000
Santa Cruz, sc-293	XPB	1/1000	1/1000
MBL international, TDM-2	CPD	N.A.	1/1000
Novus Biologicals, NBP2-38556	GTF2H1	1/500	N.A.
Santa Cruz, sc-853	XPA	1/250	N.A.
Bethyl, A301-484A	XPG	1/1000	N.A.
Enzo, BML-PW8810	FK2	1/1000	N.A.
Novus Biologicals, NB120-495	CSN5	1/2000	N.A.
Abcam, ab72548	CUL4A	1/1000	N.A.
Abcam, ab9194	DDB1	1/1000	N.A.
Abcam, Ab290	GFP	1/1000	N.A.
Sigma Aldrich, B512	Tubulin	1/10000	N.A.

Supplementary Table 2. Secondary Antibody list and working dilutions.

Source, Reference	Antibody	Dilutions	
		Immunoblotting	Immunofluorescence
Sigma, sab4600215	Anti-rabbit, CF IRDye 770	1/10000	N.A.
Sigma, sab4600200	Anti-rabbit, CF IRDye 680	1/10000	N.A.
Sigma, sab4600214	Anti-mouse, CF IRDye 770	1/10000	N.A.
Sigma, sab4600199	Anti-mouse, CF IRDye 680	1/10000	N.A.
Invitrogen, A11034	Anti-rabbit, Alexa Fluor 488	N.A.	1/1000
Invitrogen, A21429	Anti-rabbit, Alexa Fluor 555	N.A.	1/1000
Invitrogen, A11001	Anti-mouse, Alexa Fluor 488	N.A.	1/1000
Invitrogen, A21424	Anti-mouse, Alexa Fluor 555	N.A.	1/1000
