# Supplementary material for: Ubiquitin and TFIIH-stimulated DDB2 dissociation drives DNA damage handover in nucleotide excision repair
Source: Nat Commun. 2020 Sep 28;11:4868. doi: 10.1038/s41467-020-18705-0 (PMC7522231; doi:10.1038/s41467-020-18705-0)
Supplement: Supplementary file 3 — Reporting Summary [file 41467_2020_18705_MOESM3_ESM.pdf]

nature research

Corresponding author(s): Hannes Lans

Last updated by author(s): Aug 27, 2020

Reporting Summary

Nature Research wishes to improve the reproducibility of the work that we publish. This form provides structure for consistency and transparency in reporting. For further information on Nature Research policies, see our Editorial Policies and the Editorial Policy Checklist.

Statistics

For all statistical analyses, confirm that the following items are present in the figure legend, table legend, main text, or Methods section.

n/a	Confirmed	
☐	☒	The exact sample size (*n*) for each experimental group/condition, given as a discrete number and unit of measurement
☐	☒	A statement on whether measurements were taken from distinct samples or whether the same sample was measured repeatedly
☐	☒	The statistical test(s) used AND whether they are one- or two-sided<br>*Only common tests should be described solely by name; describe more complex techniques in the Methods section.*
☒	☐	A description of all covariates tested
☐	☒	A description of any assumptions or corrections, such as tests of normality and adjustment for multiple comparisons
☐	☒	A full description of the statistical parameters including central tendency (e.g. means) or other basic estimates (e.g. regression coefficient) AND variation (e.g. standard deviation) or associated estimates of uncertainty (e.g. confidence intervals)
☐	☒	For null hypothesis testing, the test statistic (e.g. *F*, *t*, *r*) with confidence intervals, effect sizes, degrees of freedom and *P* value noted<br>*Give P values as exact values whenever suitable.*
☒	☐	For Bayesian analysis, information on the choice of priors and Markov chain Monte Carlo settings
☒	☐	For hierarchical and complex designs, identification of the appropriate level for tests and full reporting of outcomes
☒	☐	Estimates of effect sizes (e.g. Cohen's *d*, Pearson's *r*), indicating how they were calculated

Our web collection on statistics for biologists contains articles on many of the points above.

Software and code

Policy information about availability of computer code

Data collection	Microscopy data was obtained using commercially available Leica LAS AF software or Carl Zeiss LSM software, as indicated.
Data analysis	Data was analyzed by Leica LAS AF (version 2.7.4.10100) and LAS X (version 3.5.6.21594) software, Carl Zeiss LSM (version 14.0.0.0), ImageJ/Fiji software (version 1.52p) and further processed in Excel (2016) and Prism (version 8.21).

For manuscripts utilizing custom algorithms or software that are central to the research but not yet described in published literature, software must be made available to editors and reviewers. We strongly encourage code deposition in a community repository (e.g. GitHub). See the Nature Research guidelines for submitting code & software for further information.

Data

Policy information about availability of data

All manuscripts must include a data availability statement. This statement should provide the following information, where applicable:

- Accession codes, unique identifiers, or web links for publicly available datasets
- A list of figures that have associated raw data
- A description of any restrictions on data availability

Source data underlying Figs. 1-6 and all Supplementary Figs. are provided as a Source Data file with this paper. Any other data are available from the corresponding author upon reasonable request.

Field-specific reporting

Please select the one below that is the best fit for your research. If you are not sure, read the appropriate sections before making your selection.

[x] Life sciences   [ ] Behavioural & social sciences   [ ] Ecological, evolutionary & environmental sciences

For a reference copy of the document with all sections, see nature.com/documents/nr-reporting-summary-flat.pdf

Life sciences study design

All studies must disclose on these points even when the disclosure is negative.

Sample size	sample sizes are specified in the legend to each figure and were chosen based on prior extensive experience in the applied techniques (Ribeiro-Silva 2018, PMID: 30287812; Sabatella 2018, PMID: 30165384; Slyskova 2018, PMID: 30137419; van Cuijck 2015, PMID: 26151477; Aydin, 2014, PMID: 24990377; Dinant 2013; PMID: 23973375), which allows us to estimate beforehand how many samples should be analyzed to provide sufficient statistical power to distinguish real differences.
Data exclusions	no data were excluded
Replication	Experiments were replicated two to five times, as indicated per experiment in the legends. all replication attempts were successful
Randomization	this is not applicable as our sample groups (cells growing in a dish) are not individually labeled and therefore automatically randomized when given treatment or not
Blinding	blinding was not needed as data is collected by imaging software which yield unbiased, objective measurements

Reporting for specific materials, systems and methods

We require information from authors about some types of materials, experimental systems and methods used in many studies. Here, indicate whether each material, system or method listed is relevant to your study. If you are not sure if a list item applies to your research, read the appropriate section before selecting a response.

Materials & experimental systems

n/a	Involved in the study	
[ ]	[x]	Antibodies
[ ]	[x]	Eukaryotic cell lines
[x]	[ ]	Palaeontology and archaeology
[x]	[ ]	Animals and other organisms
[x]	[ ]	Human research participants
[x]	[ ]	Clinical data
[x]	[ ]	Dual use research of concern

Methods

n/a	Involved in the study	
[x]	[ ]	ChIP-seq
[x]	[ ]	Flow cytometry
[x]	[ ]	MRI-based neuroimaging

Antibodies

Antibodies used	antibodies are listed in Tables S1 and S2
Validation	specificity of all our used antibodies was validated by western blot and/or IF analysis, by siRNA depletion or knockout of the target protein, or by co-IP experiments. Specifically the following antibodies were used:<br>Abcam, ab181136, rabbit anti-DDB2, knockout validated, immunoblotting and immunofluorescence, in Fig 5a and Suppl Fig. 3a<br>Bethyl, A301-121A, rabbit anti-XPC, immunoblotting and immunofluorescence, in Suppl Fig 2e and 4c<br>Santa Cruz, sc-293, rabbit anti-XPB, immunoblotting and immunofluorescence, in Fig 4e and Suppl Fig 3c<br>MBL international, TDM-2, mouse anti-CPD, immunofluorescence, in Fig 2.<br>Novus Biologicals, NBP2-38556, rabbit anti-GTF2H1, verified on a Protein Array, immunoblotting and immunofluorescence in Suppl Fig 1c and h<br>Santa Cruz, sc-853, rabbit anti-XPA, immunoblotting in Suppl Fig 1f and g<br>Bethyl, A301-484A, rabbit anti-XPG, immunoblotting in Supll Fig 1d<br>Enzo, BML-PW8810,FK2, mouse, Recognizes mono- and polyubiquitinylated protein conjugates in a wide range of species. Immunoblotting in Suppl Fig 2f<br>Novus Biologicals, NB120-495, mouse anti-CSN5, immunoblotting in Fig 6c<br>Abcam, ab72548, rabbit anti-CUL4A, immunoblotting in Fig 6c<br>Abcam, ab9194, goat anti-DDB1, immunoblotting in Fig 6c<br>Abcam, Ab290, rabbit anti-GFP, Anti-GFP antibody (ab290) is a highly versatile antibody that gives a stronger signal than other anti-GFP antibodies available. On Western blot the antibody detects the GFP fraction from cell extracts expressing recombinant GFP fusion proteins and has also been shown to be useful on mouse sections fixed with formalin, immunoblotting in Fig 6c

Sigma Aldrich, B512, mouse anti-Tubulin, immunoblotting in Fig 3d and f

Eukaryotic cell lines

Policy information about cell lines

Cell line source(s)	U2OS and MRC-5 from ATCC<br>HCT116 from Horizon Discovery<br>XP4PA described in PMID: 3029584, gift from Alain Sarasin<br>VH10 described in PMID: 23045548 , gift from Leon Mullenders
Authentication	none were authenticated
Mycoplasma contamination	all cell lines tested negative for mycoplasma
Commonly misidentified lines (See ICLAC register)	no commonly misidentified cell lines were used in the study.
