# Supplementary figures and images for: Ubiquitin and TFIIH-stimulated DDB2 dissociation drives DNA damage handover in nucleotide excision repair
Source: Nat Commun. 2020 Sep 28;11:4868. doi: 10.1038/s41467-020-18705-0 (PMC7522231; doi:10.1038/s41467-020-18705-0)

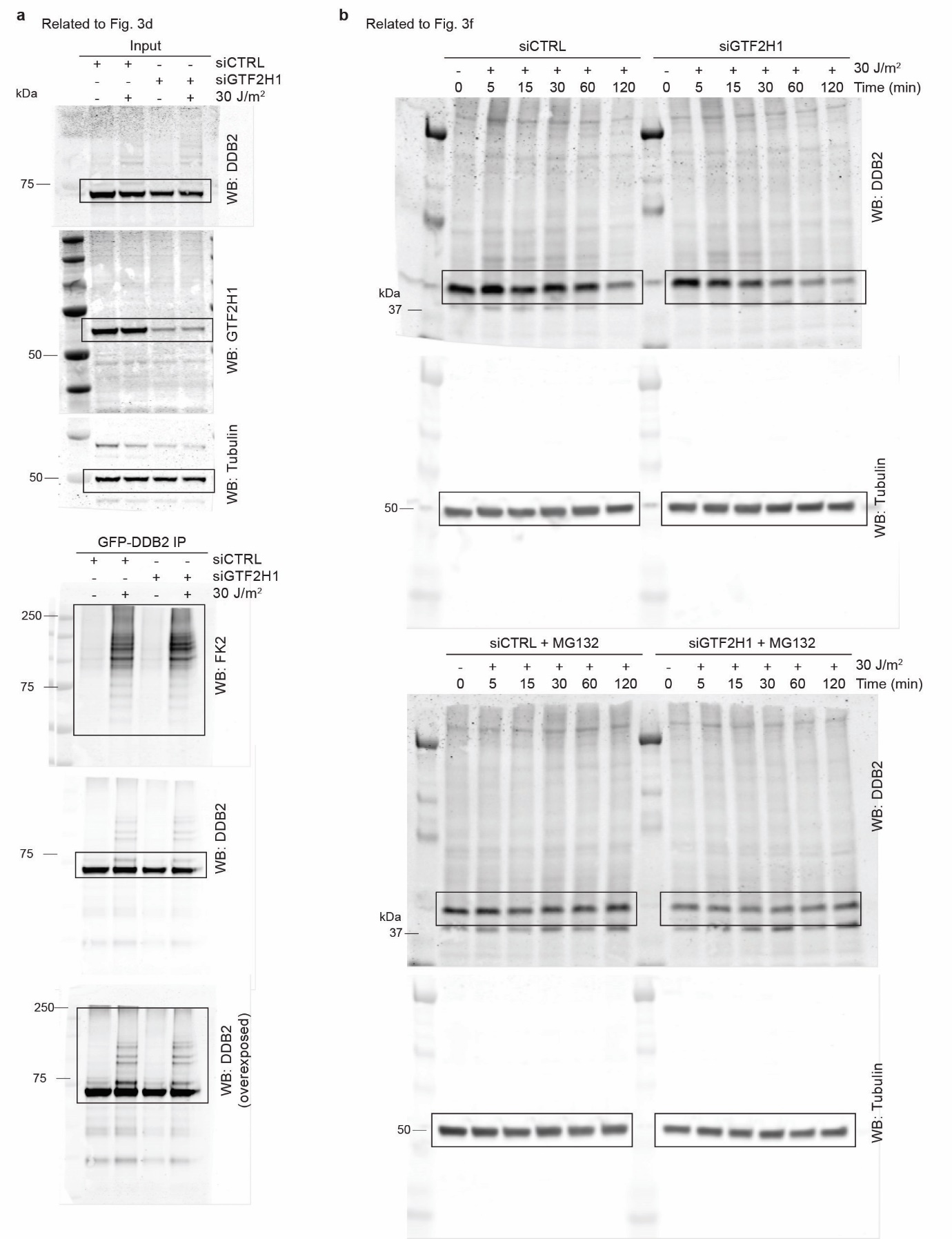


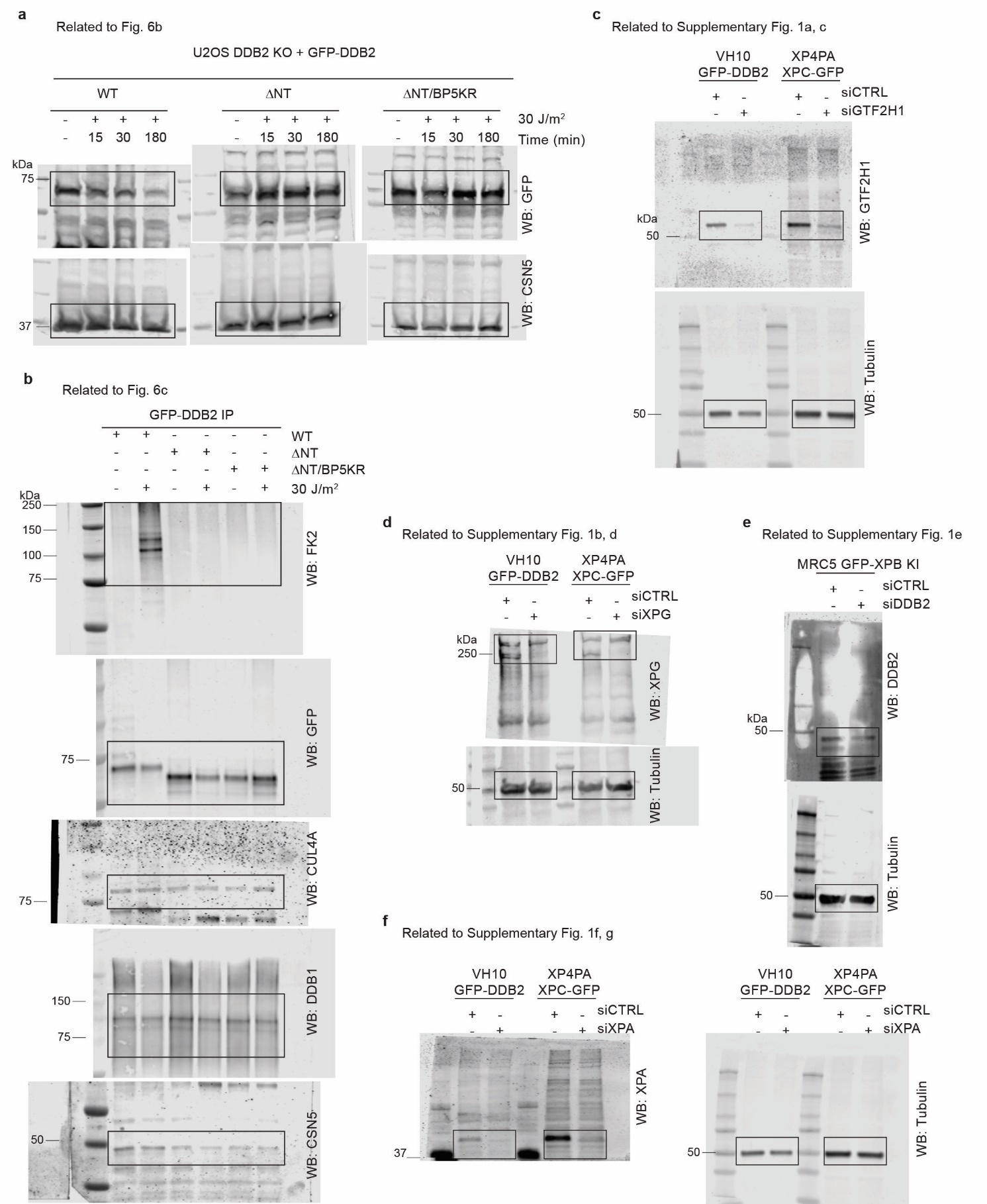


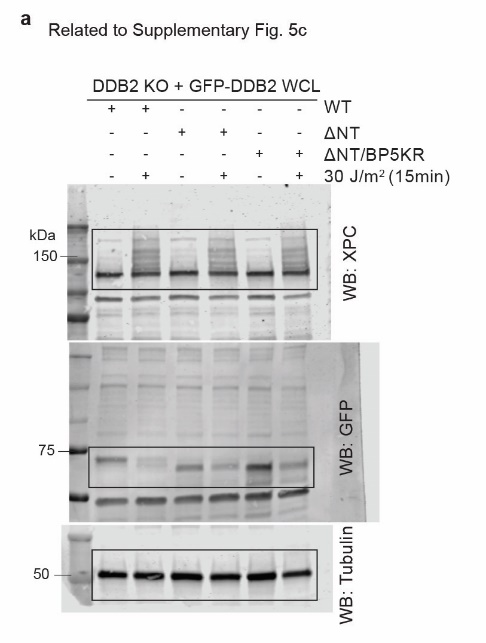

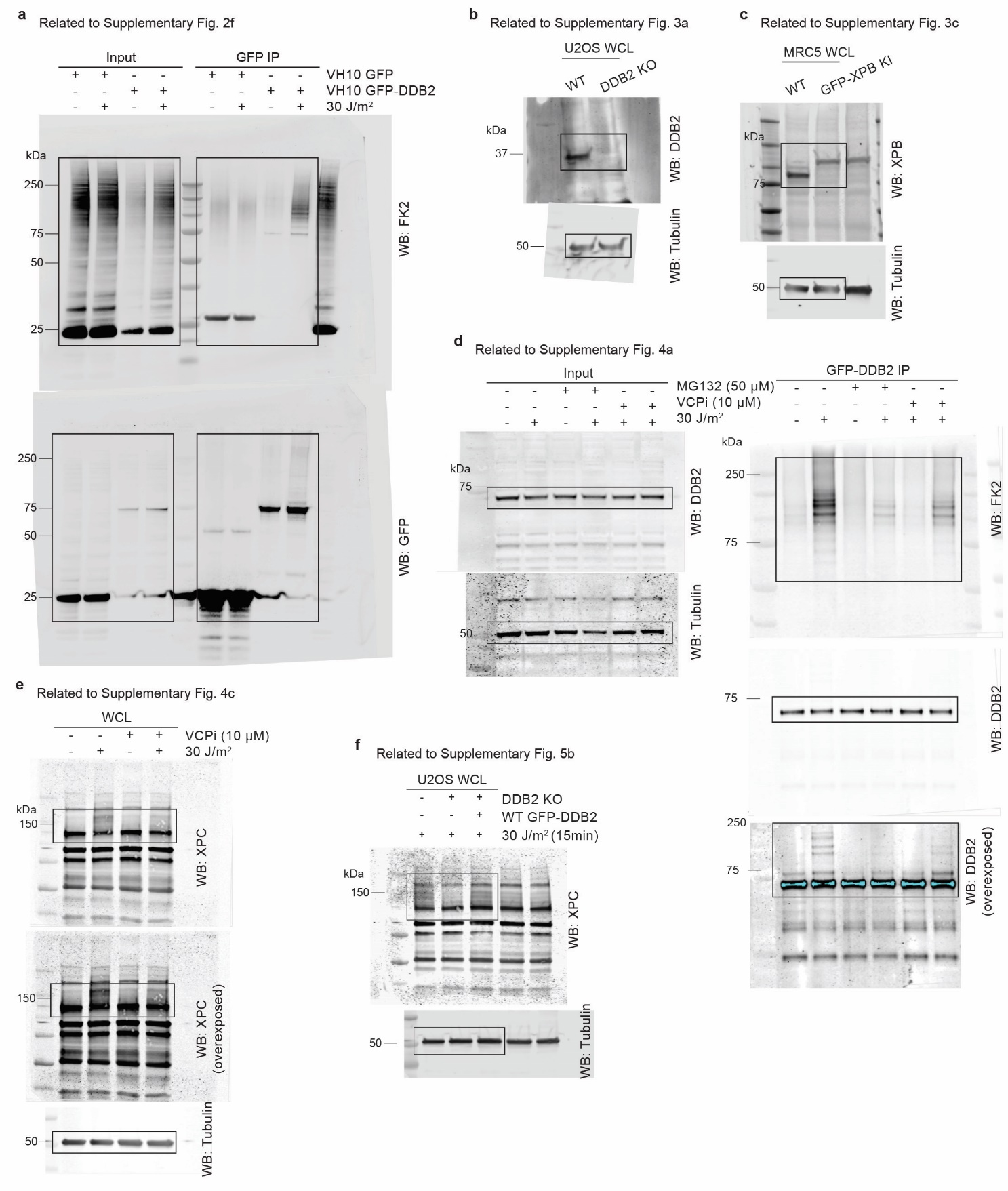

Supplement: Supplementary file 4 — Source Data [file 41467_2020_18705_MOESM4_ESM.zip › Source data file 2_wbs.docx]
